# Quantitative Assessment of Facial Paralysis Using Dynamic 3D Photogrammetry and Deep Learning: A Hybrid Approach Integrating Expert Consensus

**DOI:** 10.3390/s25113264

**Published:** 2025-05-22

**Authors:** Xiangyang Ju, Ashraf Ayoub, Stephen Morley

**Affiliations:** 1 Medical Devices Unit, Department of Clinical Physics and Bioengineering, NHS Greater Glasgow and Clyde, Glasgow G3 8SJ, UK; 2 Dental School, MVLS College, University of Glasgow, Glasgow G12 8QQ, UK; ashraf.ayoub@glasgow.ac.uk; 3 Canniesburn Plastic Surgery Unit, Royal Infirmary of Glasgow, Glasgow G4 0SF, UK; stephen.morley@nhs.scot

**Keywords:** facial paralysis, dynamic 3D photogrammetry, machine learning, PointNet

## Abstract

**Highlights:**

PointNet trained with preprocessed point clouds of facial movements can provide accurate assessment of facial paralysis.

**What are the main findings?**

- The accuracy of the new approach was higher than 95%.
- The objective approach offers better assessment than the subjective approach.

**What are the implications of the main findings?**

- The automated objective assessment of facial paralysis is achievable.
- The deep learning approach enhanced dynamic 3D photogrammetry for facial paralysis assessment.

**Abstract:**

The subjective assessment of facial paralysis relies on the expertise of clinicians; the main limitation is intra-observer and inter-observer reproducibility. In this paper, we proposed a deep learning approach combining point clouds of facial movements with expert consensus to objectively quantify the severity of facial paralysis. A dynamic 3D photogrammetry imaging system was used to capture the facial movements of five facial expressions. Point clouds of the face at rest and at maximum expressions were extracted. These were integrated with the experts grading of the severity of facial paralysis to train a PointNet network to quantify the severity of facial paralysis. The results showed an accuracy exceeding 95% for assessing facial paralysis.

## 1. Introduction

Facial paralysis is a common disorder of the facial nerves, causing weakness and the disability of facial expressions. The patients lose control of the affected side of their face, leading to the drooping or stiffness of facial muscles. Facial paralysis can not only cause significant facial asymmetry, but it can also affect eyesight when the protective closure mechanisms are lost, leading to corneal ulceration.

Current assessments of facial paralysis in clinical practice rely on the observation of patients performing specific voluntary facial expressions to assess the function of facial muscles. The clinicians subjectively graded the abnormalities in facial morphology and muscle movements. Subjective facial paralysis grading scale systems, such as the House-Brackmann scale [1], Sunnybrook Grading scale [2], and Yanagihara system [3], were used to grade the severity of facial paralysis. Fattah et al. [4] reviewed various facial paralysis grading scales. They concluded that the Sunnybrook Facial Grading was the only scale that satisfied all criteria, which included the convenience of clinical use, providing a regional scoring, static and dynamic measures, and unique features secondary to facial palsy (e.g., synkinesis). The subjective approaches of assessing facial muscle’s function presented the shortcoming of low inter-observer and intra-observer reproducibility and had limited sensitivity and specificity.

In order to improve the diagnosis and management of facial paralysis, the clinicians need an objective approach to quantify the functional abnormalities associated with facial muscle movements. Various objective approaches were used to achieve this. Standard facial images [5,6,7,8,9,10], videos [11,12,13,14], and 3D and 4D images [15,16,17,18,19,20,21,22,23,24] have been proposed to assist clinicians to quantify the severity of facial paralysis. Sophisticated Moiré pattern [25] and facial blood flow images [26] were proposed to enhance particular facial features for the diagnosis of facial paralysis. Some recent review papers [27,28,29] are more inclusive than previous published papers. So far, no objective assessment methods are widely accepted for the routine clinical assessment of facial paralysis.

Approaches based on 2D images and videos are readily available using off-shelf cameras. The 2D analysis of facial muscle movements is limited. Strey et al. [30] attempted to generate 3D models from 2D images, but errors in the various approaches to generating 3D models were notable. It was reported that 2D analysis underestimated 3D facial movement amplitudes by up to 43% [31]. 3D imaging approaches provided more accurate analysis of the 3D facial shape morphology and facilitated the tracking of facial landmarks or surface points throughout the sequence of the captured 3D facial images.

In order to assess the severity of facial paralysis, facial asymmetry both at rest and when moving was considered in most objective grading scales of facial paralysis [7,9,12,13,21,24,26,32,33]. This is not surprising as facial asymmetry is a significant feature of facial paralysis. Regional and whole-face analysis became more popular compared to landmark-based analysis for the detection and assessment of facial paralysis, especially when the machine learning approaches were applied on images or videos [7,12,13,32,33]. Only using landmarks or key points on the face required the more accurate marking or detection of these specific points.

Advances in deep learning and their expanding applications have been utilised to assess facial paralysis. Image-based approaches [11,34] showed an accuracy of up to 98% in the detection of facial paralysis; video-based methods produced an accuracy of 95% [14]; and 3D imaging improved the accuracy of classification of facial paralysis by 82% [24]. PointNet [35,36] demonstrated that 3D point clouds can be trained to improve the accuracy of the recognition of 3D facial expression.

In this paper, we presented our study on the application of PointNet to objectively quantify the severity of facial paralysis.

## 2. Materials and Methods

### 2.1. Data Acquisition

The voluntary facial expressions of 16 patients with unilateral facial paralysis and 16 healthy participants were captured using a dynamic 3D stereophotogrammetry device, the Di4D capture system (Dimensional Imaging Limited, Hillington Park Innovation Centre, 1 Ainslie Road, Glasgow, G52 4RU, UK). Five voluntary expressions were recorded, which included eyebrow raising, eye closure, maximal smiling, cheek puffing, and lip puckering. The facial expression at rest was also captured. The imaging system consisted of two grey-scale cameras (1600 × 1200 pixels) and one colour camera that captured videos at 60 fps. The system was developed by combining stereo matching and optical flow techniques, being capable of reconstructing a 3D facial model at each frame and tracking any points on the face of the sequences of expressions [37].

A generic facial mesh of 7859 vertices was conformed to the 3D facial model of the first frame reconstructed from the captured stereo videos, where the conformed mesh deformed to the facial shape and maintained the topology of the generic mesh [38,39]. The vertices of the conformed face mesh were then tracked along the subsequent frames to measure the movements of the whole face. Using the conformed mesh for facial movement tracking, the correspondence between all 3D models of the sequences of expressions was established, enabling further statistical morphometric analysis.

### 2.2. Feature Engineering

The proposed PointNet network was trained on point clouds of facial expressions and the corresponding Sunnybrook grades of facial paralysis to predict the severity of facial paralysis.

The 3D facial models at rest and the maximum frames of each expression were extracted from the tracked facial expression sequence. At first, the facial model at rest was aligned with the generic facial mesh using the partial Procrustes method, where its coordinates were defined as x-axis from left to right, y-axis from foot to head, and z-axis from the back to the front [40]. Then, the facial model at the maximum expression was rigidly aligned to the 3D model at rest using three facial landmarks (the inner corners of eyes and the tip of nose). This eliminated the unwanted effect of head movements on the tracked facial movements. The coordinates of the corresponding vertices of the 3D conformed mesh of the facial models at rest and maximum expressions were the point clouds data used for the training of the PointNet network.

We used a modified Sunnybrook grading scale (Table 1) to grade the facial paralysis of each patient. First, three parameters were assessed at rest, and the other five parameters were evaluated for each of the five expressions. Seven experts assessed the severity of the facial paralysis of the recorded videos of the 3D expressions of each patient, and the grading was repeated after 45 days.

The accuracies of the assessments of individual parameters of the modified Sunnybrook grades were analysed in a previous study [22]. The 7 assessors graded 16 patients twice. The modes of the grades were calculated from 14 observations for each patient and the occurrences of the corresponding modes were obtained. The accuracy of each subjective parameter was calculated as the number of its occurrences divided by 14.

The consensus among the seven experts (modes of the grades) were used as the grades for the training of the PointNet network. The modes of the scores marked by the clinicians were used as a good standard to evaluate the accuracy of the proposed objective assessment approach.

### 2.3. Network Architecture

**Figure 1:**
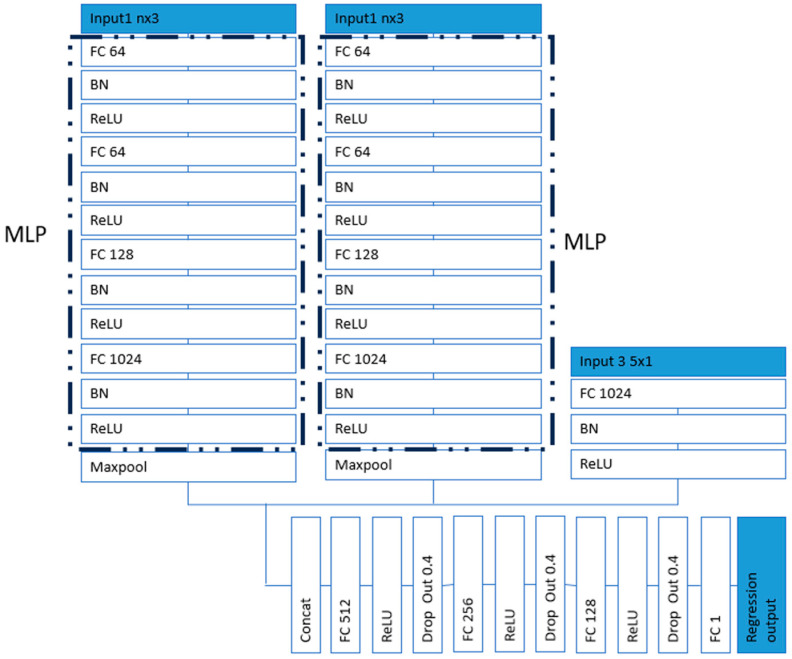
Network architecture (FC: fully connected layer, BN: batch normalisation layer).

The network architecture is shown in Figure 1, with FC representing the fully connected layer and BN representing the batch normalisation layer. Features were extracted by the PointNet layers from the point clouds (vertices of 3D facial models) at rest and at the maximum frames of each expression. MLP was used to transform individual point features and also to aggregate global features. Specifically, a shared MLP mapped each point’s input to a higher-dimensional space [35,36]. This allowed PointNet to learn the feature representations for each point independently and identically. Two parallel MLPs were used to process input 1—rest face—and input 2—maximum expression face. Each point cloud provided distinct information that was processed separately to extract relevant features. Two parallel MLPs allowed each MLP to specialise in learning the unique characteristics of each point cloud. Input 3 was a categorical type of data which provided information on the expression types. The features in combination with the expression type were connected to fully connected layers to predict the severity of facial paralysis. The output of the regression layer was the numerical grade of the expression to be assessed. It contained scores for each of the 5 expressions. An adaptive moment estimation (Adam) solver [41,42] was used for training the network. The loss function of the regression layer was the half mean squared error of the predicted responses. There were 160 original data sets which included 5 expressions for 16 patients and 16 health participants. The point clouds were randomly rotated to the left or right within 15°, and 2% of random noise was added to augment the data to 960 samples. A total of 768 samples were used for training and 192 for testing.

### 2.4. Facial Movement Observation

The conformed 3D model at each frame can also be aligned using the partial Procrustes method to its own mirrored model to detect the discrepancies between the left and right sides of the face [43,44], to quantify the facial asymmetry of each 3D captured frame. The average displacements of facial movements from the rest expression to maximum expression was calculated. The facial models of the left-sided facial paralysis were all reflected, so the paralysis was on the right side for the entire sample. The average asymmetries at maximum expressions were calculated by measuring the distances between the corresponding vertices of the conformed meshes and their mirrored copy. In perfect symmetry, this measurement would be equal to zero.

## 3. Results

**Figure 2:**
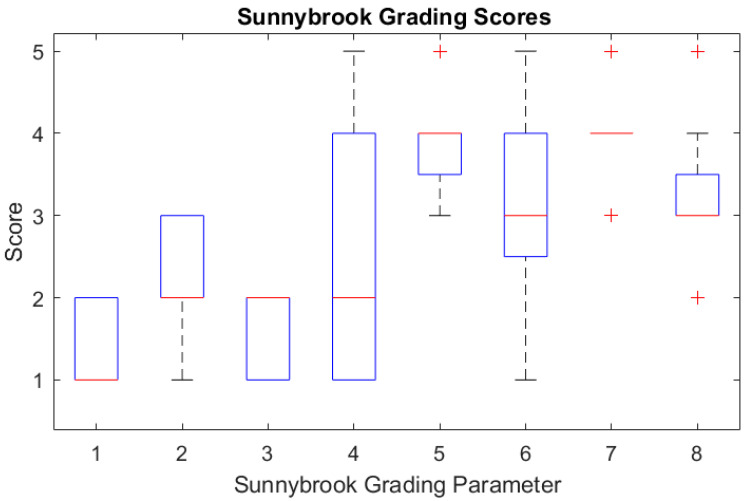
Box plot of the Sunnybrook grading scores of the 16 patients (the outliers are plotted individually using the ‘+’ marker symbol).

Sixteen patients with unilateral facial paralysis were recruited for this study, seven males and nine females with the average age of 45.3 (23–64) years old. Sixteen age-matched participants were recruited as well. The Sunnybrook grading scores of the 16 patients were box plotted and are shown in Figure 2.

**Figure 3:**
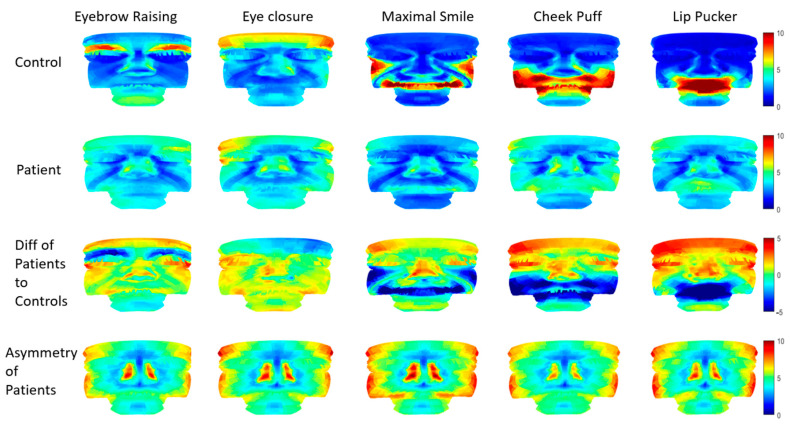
Average facial movements (control−first row; patient−second row); differences between patients (third row) and controls; and the average asymmetry of the patients (fourth row).

The average facial movements of the five facial expressions from the rest frame to the maximum frame were calculated; the average facial movements of the controls are shown in the first row of Figure 3, and that of the patients are shown in the second row. The colour map from blue to red indicates the distances from 0 mm to 10 mm. The differences between the average movements of the patients and those of the controls are shown in the third row (blue to red indicate distance differences from −5 mm to 5 mm). The average of the asymmetries of the patients across the five expressions are shown in the last row (blue to red indicate distance differences from 0 mm to 10 mm).

**Figure 4:**
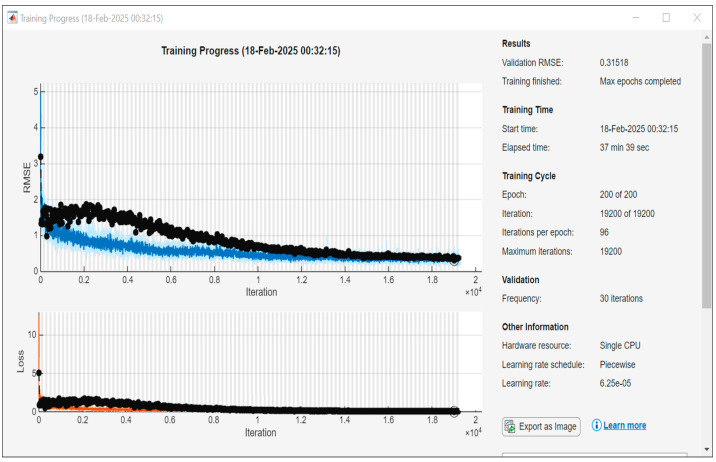
PointNet network training curves of RMSE and Loss.

**Figure 5:**
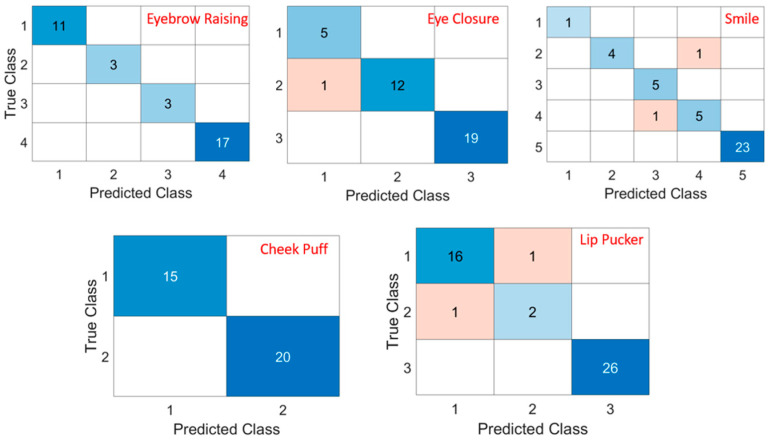
Confusion matrix of each expression.

The PointNet network was trained in Matlab R2024a with 768 samples, 200 epochs, and 96 iteration per epoch. The training finished with a mean squared error (MSE) 0.10, R-squared 0.93. The training took 38 min on a laptop of single i7 CPU. The curves of the RMSE and loss of the training process are shown in Figure 4. The 192 test results were grouped based on the types of expressions and compared to the grades of the exports. When the difference between the prediction and the grade was less than 0.5, the prediction was regarded as correct. The accuracies of the PointNet network predictions on the five expressions were higher than 95% (Table 2). The confusion matrix of each expression is shown in Figure 5. The accuracies of the Sunnybrook grading of the experts were calculated from the repeated assessments of the seven assessors as well for comparison.

## 4. Discussion

The 3D dynamic photogrammetry imaging system is capable of tracking full facial movements [37,42], and the conformation process establishes the correspondences between all the 3D models of the sequence of the 180 images captured throughout the course of each facial expression [35,40]. These facial movement data enabled statistical morphometrical shape analysis. In our previous study on facial paralysis, regional measurements were calculated to investigate the correlations between the measured asymmetries and the Sunnybrook grades. Our findings showed that the correlations between the measured asymmetries and the Sunnybrook grades were poor for cheek puffing and forceful eye closure [20]. Furthermore, shallow networks were applied on the regional asymmetries to objectively assess the facial paralysis with reported accuracies of 81.7%, 87.1%, 66.5%, 91.1%, and 77.7% for the five facial expressions, respectively [22]. The regional analysis simplified the processing of facial data by focusing on anatomically relevant regions for analysis. This included analysing lid closure in the eye region and smiling in the mouth region; but in the case of facial paralysis, this approach excluded most compensation facial movements that are outside the defined anatomical regions. In Figure 3, we can clearly see the controls performed (red regions) eyebrow raising, smiling, cheek puffing, and lip puckering but not eye closure due to the poor capturing of the stereophotogrammetry on the reflective surface of the cornea. The performance of the five expressions was weakened on the affected right side of facial paralysis, which could be seen from the differences in the blue and red colours in the third row of Figure 3. The compensation movements of the facial muscles can be seen, especially in the cheek puffing and lip puckering expressions. The asymmetries also reflect the weakening side of the face (yellow to red colours). The colour map is symmetrical due to the fact that the absolute differences between the left and right are identical.

Given that both the voluntary facial movements and the involuntary compensation movements “synkinesis” were both important in assessing the severity of facial paralysis, we believe that full facial movements analysis could improve the accuracy of the assessment. Machine learning approaches applied on full facial images [24,25] or videos [26] have achieved 98% accuracy in the detection/classification of facial paralysis. The combination of the feature extraction using the 3D dynamic photogrammetry system with the PointNet network enabled us to achieve accuracies higher than 95% for the assessment of facial paralysis based on five facial expressions. We believe that the established corresponding point cloud data enhanced the PointNet network to provide a more accurate assessment of facial paralysis.

It would be better to integrate the facial image data into the network training to enhance the performance of the network. The 3D sequence data process was time consuming and needs to be addressed.

Further work is needed to extend the assessment on all the parameters of the Sunnybrook grading scales and the other grading scales to establish an automated assessment system for the clinical assessment of facial muscle movements. We recommend a larger sample size to include all spectrums of facial paralysis for deep learning.

## 5. Conclusions

We enhanced a dynamic 3D stereo photogrammetry imaging system with the proposed PointNet network for the quantification of the severity of facial paralysis and obtained accuracies exceeding 95% for severity assessments based on five dynamic facial expressions.

## Figures and Tables

**Table 1:** Modified Sunnybrook grading system.

Parameter	Observation	Grade
Resting Symmetry Score		
Eye	Abnormal/normal	1/2
Cheek [nasolabial]	Absent/altered/normal	1/2/3
Mouth [drooped]	Abnormal/normal	1/2
Voluntary movement Score		
Forehead wrinkling	No movement—normal	1–5
Gentle eye closure	No movement—normal	1–5
Open mouth smiling	No movement—normal	1–5
Cheek puffing	No movement—normal	1–5
Lip puckering	No movement—normal	1–5

**Table 2:** Accuracy of the assessor and PointNet network on five expressions.

Expressions	Eyebrow Raising	Eye Closure	Smiling	Cheek Puffing	Lip Puckering
Assessor	74.1%	79.9%	65.2%	73.2%	73.2%
PointNet	100%	97.3%	95.0%	100%	95.7%

## Data Availability

Data are unavailable due to privacy or ethical restrictions.
